# Colouration in amphibians as a reflection of nutritional status: The case of tree frogs in Costa Rica

**DOI:** 10.1371/journal.pone.0182020

**Published:** 2017-08-24

**Authors:** Andrea Brenes-Soto, Ellen S. Dierenfeld, Geert P. J. Janssens

**Affiliations:** 1 Laboratory of Animal Nutrition, Faculty of Veterinary Medicine, Ghent University, Merelbeke, Belgium; 2 Animal Science Department, University of Costa Rica, San José, Costa Rica; 3 Ellen S. Dierenfeld, LLC, St. Louis, Missouri, United States of America; University of Sao Paulo, BRAZIL

## Abstract

Colouration has been considered a cue for mating success in many species; ornaments in males often are related to carotenoid mobilization towards feathers and/or skin and can signal general health and nutrition status. However, there are several factors that can also link with status, such as physiological blood parameters and body condition, but there is not substantial evidence which supports the existence of these relationships and interactions in anurans. This study evaluated how body score and blood values interact with colouration in free-range *Agalychnis callidryas* and *Agalychnis annae* males. We found significant associations between body condition and plasmatic proteins and haematocrit, as well as between body condition and colour values from the chromaticity diagram. We also demonstrated that there is a significant relation between the glucose and plasmatic protein values that were reflected in the ventral colours of the animals, and haematocrit inversely affected most of those colour values. Significant differences were found between species as well as between populations of *A*. *callidryas*, suggesting that despite colour variation, there are also biochemical differences within animals from the same species located in different regions. These data provide information on underlying factors for colouration of male tree frogs in nature, provide insights about the dynamics of several nutrients in the amphibian model and how this could affect the reproductive output of the animals.

## Introduction

Animals that have the capacity to invest their resources in survival and reproductive success are favoured evolutionarily. Visual signals, such as coloured ornamentation in feathers and skin, are used as cues for mating choice in several species [1]. Many colourful ornaments are due to the presence of carotenoids, which are acquired by animals only through the diet [2]. Carotenoids are also important in antioxidant and immune defences [1], and play a relevant role in vitamin A synthesis due to provitaminic activity [3]. Beta-carotene provides a major source of vitamin A activity, although it has been reported that some xanthophylls can act as precursors of vitamin A in fish as well as in amphibians [4–7]. Likewise, dietary carotenoid supplementation has been directly linked with circulating carotenoid concentrations, growth, and reproductive outputs [7,8,9].

Carotenoid pigments associated with colouration involve nutritional costs for acquisition as well as for utilization [10,11]. Healthier animals in good body condition thus appear to accumulate carotenoids to maximize ornamental display, having already met their primary physiological needs of pigments for immune and antioxidant response. Only individuals with access to dietary pigments in excess of those needed for health and nutritional functions can afford the immunologic costs of investing carotenoids in ornaments, using this resource to enhance sexual display [1,11,12]. The preference of females for more ornamented males indicates the choice for phenotypic quality linked with direct or indirect genetic benefits. Stronger ornamentation can reflect the ability of the male to provide material advantages, such as fertility, high quality territory, nutrition, protection and the maintenance of the genetic variation [13,14]. Mate choice studies in fish and birds determined that those individuals displaying better carotenoid pigmentation in sexual signals are preferred over others [15,16].

In amphibians, pigmentation can influence courtship and mate selection, affecting potential recognition of breeding partners and perception of fitness, indirectly affecting reproductive success, resulting in fitness gains for picky females and for brightly coloured males [17,18]. Carotenoids have a wide distribution in the amphibian body, and numerous compounds have been found in the skin of several species (Table 1) [19–21]. These pigments are localized in chromatophores in the dermis and epidermis, arranged in dermal chromatophore units, namely melanophores (containing melanine), xanthophores (containing carotenoids pigments and ranging in colour from yellow to red) and iridophores (responsible for the elaboration of the green colouration by transmitting and reflecting light through the overlying xanthophores), which respond to both morphological and physiological stimuli, to induce colour changes by varying the amount as well as the dispersion or aggregation of the pigments [21,22]. In amphibians, allocation of pigments in the chromatophores is controlled by circulating levels of several hormones, but alterations are influenced by other environmental and physiological triggers [23]. The colour change mechanisms in amphibians are still unclear; there is a need for further evidence to support that amphibian colouration reflects nutritional status.

**Table 1:** Carotenoids pigments identified in the skin of several anuran species.

Species	Colour	Carotenoid	Reference
*Rhacophorus arboreus*	Blue-green	ß-carotene and other non specified	[20]
*Hyla arborea japonica* *(Dryophytes japonicus)*	Green	ß-carotene and other non specified	[20]
*Bufo (Bufo) japonicus*	Yellow-green	ß-carotene and other non specified	[20]
*Rana catesbeiana*	Green-brown	ß-carotene and other non specified	[20]
*Rana japonica*	Red-brown	ß-carotene and other non specified	[20]
*Bombina orientalis*	Red-green	ß-carotene, 4-hydroxy-echinenone	[24]
*Hyla japonica* *(Dryophytes japonicus)*	Green-yellow	Non specified	[25]
*Rana pipiens*	Green-yellow	Non specified	[26]
*Rana temporaria*	Brown-orange	Xanthophylls and other non specified	[27]
*Bombina bombina*	Brown-red	ß-carotene, ß-cryptoxanthin, lutein, zeaxanthin	[19]
*Pelobates fuscus*	Brown-orange	ß-carotene, γ-carotene, ß-cryptoxanthin, cantaxanthin, lutein, zeaxanthin	[19]
*Bufo bufo*	Brown-yellow	ß-cryptoxanthin, isocryptoxanthin, astaxanthin ester	[19]
*Rana esculenta* *(Pelophylax esculentus)*	Green-yellow	ß-carotene, ß-cryptoxanthin, canthaxanthin, astaxanthin ester	[19]
*Rana ridibunda* *(Pelophylax ridibundus)*	Green-brown	ß-carotene, ß-cryptoxanthin, lutein, astaxanthin ester	[19]

Assessment of nutritional status is a challenging task in free-living species, but across species, researchers have used morphometric analysis, comparing size and weight to estimate body condition [28,29], as well as measurement of blood metabolite concentration, the latter which may render more mechanistic insights into nutritional status [30]. Nonetheless, still there is not substantial evidence to explain how well these measurements agree with colouration. Body condition is a valuable tool to estimate muscular definition and external deposits of adipose tissue, reflecting energy reserves of the animal; therefore this system contributes to identify if diet changes are needed to improve general health [28,31]. In amphibians, some authors have found a significant relationship between body condition and colouration patterns in the red-spotted newt *Notophthalmus v*. *Viridescens* [17] and the moor frog (*Rana arvalis*), suggesting effects on mating status and fighting ability [32].

Blood analyses allow the health assessment of the animals, providing information about internal organ status, electrolytes, immunological condition as well as nutritional and metabolic parameters, and also can reflect changes in the ecological and environmental condition of a natural population [33, 34]. For several species that receive veterinary care, blood biochemistry reference ranges exist [34]. Although there are many haematological reports on anuran species, both extrinsic and intrinsic factors complicate the establishment of those ranges in free range species [35].

Any important diet shift can result in measurable changes in blood composition, and general blood and plasma values such as glucose, proteins and haematocrit can be useful indicators of such changes. In animals, blood glucose homeostasis is maintained by the equilibrium between glucose supply and removal, as a result of a finely balanced system of hormonal interactions [36]. Strict carnivores obtain glucose mainly from gluconeogenesis via aminoacid transaminationor from glycerol after lipolysis [37,38]. In the absence of problems with insulin sensitivity, the blood glucose concentration thus reflects the overall availability of the main dietary energy sources for carnivores, i.e. protein and fat [39], meanwhile plasmatic proteins further represent the protein status of the animals. In domestic animals, haematocrit often links with health status because it is an indicator of water balance in the body, also involving other dietary nutrients such as electrolytes, iron, folic acid, vitamins B_6_ and B_12_ [36,40]. Haematocrit varies due to characteristics of amphibian biology, such as water and skin solute exchange, haemodilution and haemoconcentration [41]. Knowledge of these haematological parameters and how they can be related to colouration will likely render more insight than simply body condition scores in assessing animal nutrition and health [11,42], and may provide early indications of nutrient imbalances arising from habitat changes, resource declines, and negatively impacted immune status. Though published data exist concerning blood values in some anurans, considerable variation and interspecies differences have also been reported [43], due to age, sex, weight, food type and climate [41]. Yet, in none of these studies has the link with skin colouration been investigated.

Many amphibians are very sensitive to fluctuations in environmental conditions; climate change, habitat loss/fragmentation, UV radiation, chemical contaminants, diseases, among others, are factors affecting the decline of amphibians´ populations, and which ultimately have negative consequences on animal health [44, 45]. Given the importance of colour for survival and breeding success [13], we here investigate the morphological and physiological relationship among haematological parameters, body condition and colouration in two Costa Rican colourful tree frog species, *A*. *callydrias* and *A*. *annae*, over two years to evaluate the association between nutritional cues and skin colour in wild amphibians. The red-eyed tree frog, *A*. *callidryas*, and blue-sided tree frog, *A*. *annae*, are hylids endemic to Costa Rica, with their habitats including humid forest lowlands and humid pre-montane areas respectively [46]. Both species are nocturnal and arboreal [47], consuming locally available insect prey as adults. Although *A*. *callidryas* is of less conservation concern, *A*. *annae* is listed as Threatened [48], and populations of both are declining. These species were selected for the study because of their abundance in suitable habitat [46,49] and general appropriate body size to obtain different measurements and colour readings among species as well as populations. Within Costa Rica known genetic isolation occurs between populations of *A*. *callydrias*, with patterns of gene flow interrupted by biogeographic barriers, thus genetics play an important role in the distribution of observed phenotypic differentiation, [50].

The relationships among skin colouration characteristics, concentrations of blood glucose, plasma proteins, haematocrit and body condition score in these frogs were used to evaluate underlying factors determining nutritional status in the wild.

## Materials and methods

**Fig 1:**
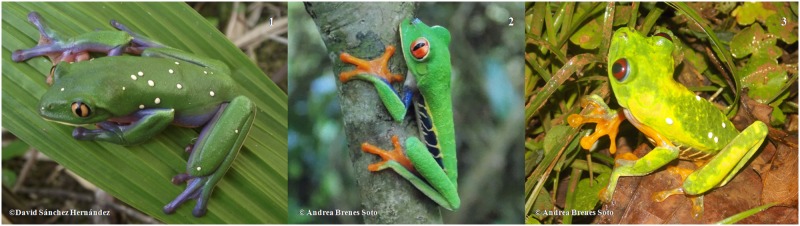
Species and groups of the study. 1: *A*. *annae*, 2: *A*. *callidryas* Atlantic population, 3: *A*. *callidryas* Pacific population.

The study was performed on two frog species, *A*. *annae*, with upper surface green, blue flanks and belly creamy yellow to orange, and *A*. *callidryas*, with upper surface generally leaf green, ventral surface creamy white and yellow [46]. Within *A*. *callidryas* species, two populations were included: the Atlantic morph with blue flank stripes and the Pacific morph with orange to brown flank stripes [51,52] (Fig 1).

Eighty six adult male frogs were collected from the wild during the breeding season (June to November) in three sites in Costa Rica over a period of two years: n = 35 *A*. *callidryas* from the Pacific population (year 1, n = 16; year 2, n = 19), site San Isidro de Dota (9°33´57.99”N, 84°05´35.12”W) in an area of 5000 m^2^; n = 31 *A*. *callidryas* from the Atlantic population (all collected in year 2), site El Zota Biological Station (10°33´43.7”N, 83°44´1.77”W) in an area of 10000 m^2^, and n = 20 *A*. *annae* (year 1, n = 11; year 2, n = 9), site San José (9°56´19.24”N, 84°04´23.68”W) in an area of 2000 m^2^. Animals were collected under Ministry of Environment and Energy permit No.05513 and procedures for this study were approved by the Institutional Committee of Use and Care of Animals of the University of Costa Rica, No. 29–11.

The collection of the animals began after 19:00 hours. Animals were found near ponds and localized by identifying the male´s song, after a period of recognition training. All the procedures for collection, handling and management of the animals were standardized in order to avoid a possible bias due to stress. Frogs were collected by hand, wearing nitrile gloves, and individually maintained during the night in 10 X 10 X 15 cm plastic containers containing a wet paper towel and a leaf. Early the following morning, after a period of 10 hours fasting, all measurements were taken *in situ*, in conditioned spaces to work, in this order: morphometrics, colouration and blood sampling. Although it is acknowledged that suggested mechanisms for colour changes in *Agalychnis dacnicolor* and *A*. *callidryas* have been described, that can be triggered by external stimuli [53,54], such changes are typically of short duration [21], and colour in this study was measured after an average of 10 hours post collection. Animals were not exposed directly to sunlight, and uniform white paper towels were used to drape the inside of the plastic container as well as the area where the measurements were taken [53].

Morphometric measurements were determined (W: weight, L: Length) using a CQT-202 Adam Equipment Company 200g (±0.01 g) scale and 180 mm Vernier caliper (±0.1 mm), and ratio was calculated (W/L) as an estimate of body condition [55]. Quantitative colouration of both ventral and dorsal surfaces was measured aiming for the centre of the area, using a hand-held spectrophotometer (Konica Minolta^®^ CM-700d, Konica Minolta Sensing Americas, Inc., Ramsey, New Jersey, USA)with a diameter of measurement of 1.2 cm. Data were then registered through the software Spectramagic NX^®^, including –a* (green), +a* (red), +b* (yellow) and L*(lightness) coordinates as well as chroma (C*) and hue of the chromaticity diagram. Chroma was calculated as indicated in the following formula: C = √(a)^2^ + (b)^2^.

Animals were anesthetized using a solution of isofluorane mixed with distilled water and ultrasound gel, applied topically at a dose of 0.03 ml/g body weight. Blood samples (3% of body weight [56]) were drawn by heart puncture using a tuberculin syringe, for determination of glucose, plasmatic proteins and haematocrit. Glucose was determined using a portable kit (Multicare in^®^, Biochemical Systems International, Florence Italy). Then blood was centrifuged using a haematocrit centrifuge (Equipslab^®^ Ningbo Equipslab International Co. Ltd., Ningbo, China) for haematocrit measurement and plasma samples were used to determine plasmatic protein with a portable refractometer (Boeco^®^, Boeckel & Co, Hamburg Germany). After total recovery (±7 hours), animals were returned to the same location as collection after 19:00 hours.

Statistical comparisons were expressed as the means and differences, and were considered significant at p<0.05. Univariate ANOVA was applied to analyse the responses between *Agalychnis* species and locations of separate *A*. *callydrias* populations, with a Tukey comparison test performed to determine differences between species/groups. All data were further combined into a higher level frog assemblage, to examine broader relationships among morphometric measurements, blood parameters and colouration. Then, principal components analysis (PCA) was carried out and was deemed important with a value above 0.5 in each component, and Pearson´s correlation coefficients were determined, both to obtain the association (s) among all the variables of the entire set of animals. All statistical analyses were conducted using the SPSS^®^ 23 program.

## Results

### Species and groups

Significant differences among populations were detected in all parameters evaluated, except the dorsal –a* axis colouration (Table 2).

**Table 2:** Average values of body condition, blood values and colouration of three groups of free ranging *Agalychnis* spp.

Parameter	*Agalychnis* *annae* (n = 20)	*Agalychnis* *Callidryas* SI (n = 35)	*Agalychnis* *Callidryas* EZ (n = 31)	*P*
**Body condition**				
**Weight (g)**	7.0±0.9^a^	5.2±0.6^b^	5.3±0.7^bc^	<0.001
**Length (mm)**	57.5±3.5^a^	48.9±2.6^b^	52.4±2.4^c^	<0.001
**Ratio W:L (g/mm)**	0.121±0.012^a^	0.106±0.010^b^	0.102±0.010^bc^	<0.001
**Blood values**				
**Blood glucose (mg/dl)**	50±12^a^	56±12^ab^	41±7^c^	<0.001
**Plasma protein (g/dl)**	4.0±1.0^ab^	4.0±0.6^a^	3.4±0.8^b^	0.023
**Haematocrit (%)**	30±7^a^	20±7^b^	18±7^bc^	<0.001
**Colour (Dorsal)**				
Lightness (L*)	60±6^a^	66±4^b^	67±5^bc^	<0.001
-a*Coordinate	-27±4^a^	-25±4^a^	-24±3^a^	0.14
+b*Coordinate	47±8^a^	54±8^b^	55±7^bc^	0.002
Chroma (C*)	54±9^a^	60±8^b^	60±6^bc^	0.016
**Hue**	120±3^a^	115±3^b^	114±5^bc^	<0.001
**Colour (Ventral)**				
Lightness (L*)	76±4^a^	80±3^b^	85±2^c^	<0.001
+a * Coordinate	10±3^a^	7±3^b^	0.2±1.5^c^	<0.001
+b * Coordinate	28±6^a^	38±6^b^	21±3^c^	<0.001
Chroma (C*)	30±6^a^	39±6^b^	21±3^c^	<0.001
**Hue**	70±5^a^	80±5^b^	90±4^c^	<0.001

Different superscripts (a,b,c) within rows differ significantly (*P*<0.05) according to Tukey test.

SI: San Isidro/Pacific population, EZ: El Zota/Atlantic population

*A*. *annae* individuals were largert han *A*. *callidryas*, and *A*. *callidryas* from the Atlantic population were larger than the Pacific group. Not only were glucose values lower in *A*. *callidryas* from the Atlantic compared to the other two groups, but significant differences were also found between Pacific and Atlantic populations of the same species. Meanwhile the protein levels varied only between the two populations of *A*. *callidryas*, and haematocrit differed only in *A*. *annae* compared to both groups of *A*. *callidryas*.

On the dorsal surface, lightness and chroma values indicated that *A*. *annae* individuals had darker and less saturated colours compared to *A*. *callidryas*. Green coordinates (-a*) values showed no differences, but yellow coordinates (+b*) differed between *A*. *annae* and *A*. *callidryas*, meaning that the differences in colour on the back in those species were mostly due to the +b* values. Hue measurements indicated that the green colour was more intense in *A*. *annae* compared to *A*. *callidryas*, and the back of *A*. *callidryas* from the Pacific, which was greener compared to the Atlantic population.

The ventral colouration also showed differences among the three groups. Colour of *A*. *callidryas* from the Atlantic was paler and less saturated than the other two groups; +a* and +b* coordinates indicated that *A*. *annae* was yellower than *A*. *callidryas* from the Pacific, which presented a slightly more orange colour; *A*. *callidryas* from the Atlantic showed a paler yellow compared to the other two groups. However, hue values from the Atlantic population indicated that the colours were more intense in that population compared with the other two (S1 Fig).

### Associations between parameters in the combined *Agalychnis* sp. data

**Fig 2:**
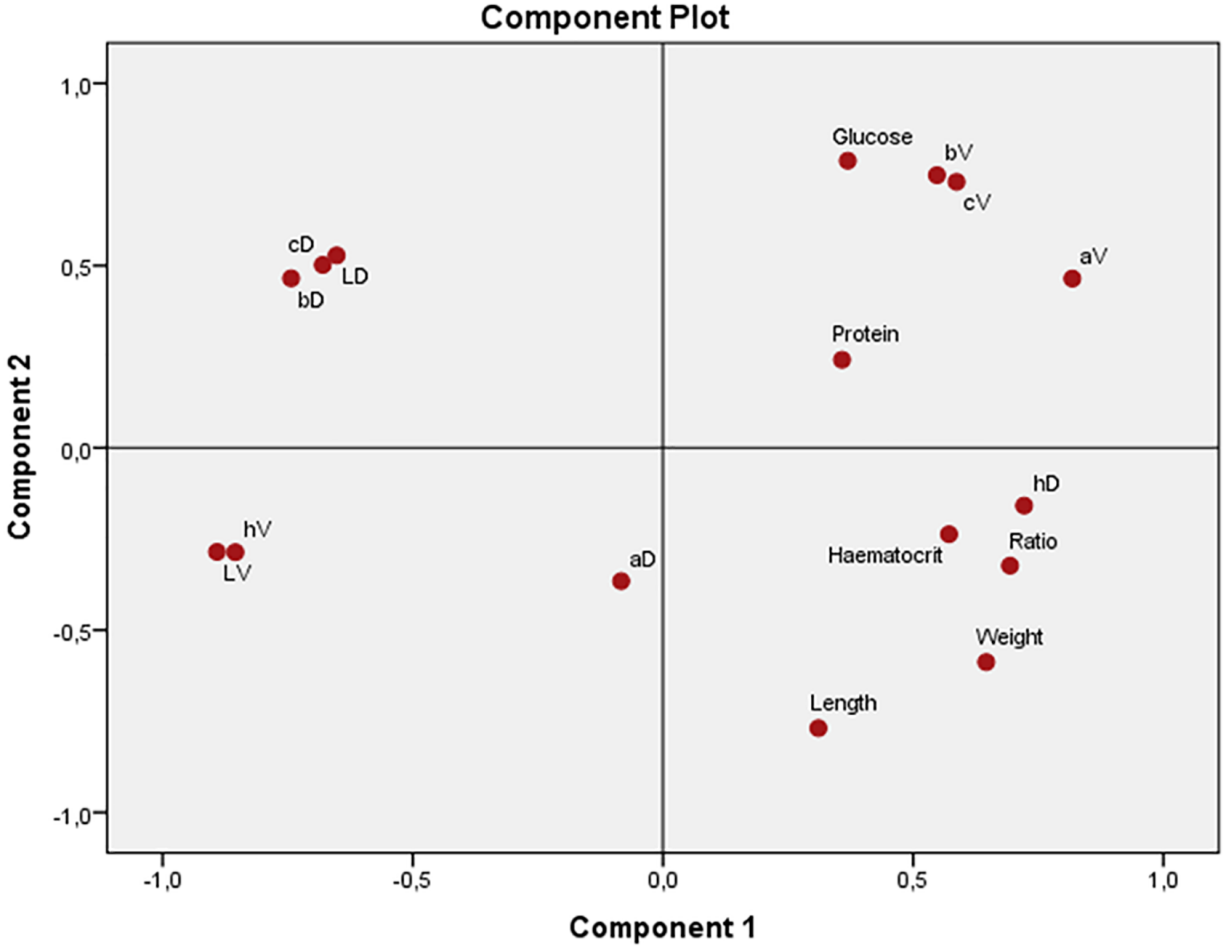
Principal components analysis (PCA) plot of body condition, blood metabolites and colouration of the combined *Agalychnis* spp. data from Costa Rica. LD: dorsal lightness, aD: Dorsal -a* coordinate, bD: dorsal +b* coordinate, cD: dorsal chroma, hD: dorsal hue. LV: ventral lightness, aV: ventral +a* coordinate, bV: ventral +b* coordinate, cV: ventral chroma, hV: ventral hue.

The PCA biplot (Fig 2) shows how variables are associated. The first principal component increases with increasing weight, ratio, haematocrit and dorsal hue, as well as most of the ventral colour values, suggesting that both haematocrit and dorsal hue tend to associates strongly with the body condition of the animals. Nonetheless, the variation of haematocrit and body condition of the animals is inversely associated with dorsal lightness as well as with the ventral values. The second principal component raises with increasing glucose, ventral chroma and +b* colour coordinate, whereas it shows an inverse association between morphometric measurements and glucose, ventral chroma and +b* value.

#### Blood values and body condition

The body condition of the frogs, expressed as the ratio between weight and length, was directly related to the level of plasmatic proteins (p = 0.010) as well as haematocrit (p<0.001) (Table 3). Likewise, there was positive relationship between glucose and protein levels (p = 0.003) as well as between protein and haematocrit (p = 0.001) (S2 Fig).

**Table 3:** Pearson´s correlations between morphometric measurements and blood values of the combined *Agalychnis* spp. data from Costa Rica.

Parameter	Glucose	Protein	Haematocrit
**Weight**	-0.009	0.259	**0.506**†
**Length**	-0.167	0.053	**0.375**†
**Ratio**	0.093	**0.339**†	**0.508**†

^**†**^Significant (*P*<0.05). Colour intensity in the table indicates the strength of the relationship between variables.

#### Body condition and colouration

Body condition measurements were inversely correlated with both dorsal and ventral lightness, +b*coordinate and chroma. Meanwhile, ventral +a* coordinates were positively related to weight and ratio. The relation was strongest in lightness, +a* coordinate and ventral hue, and the effect was overall due to the weight value (p<0.0001) (Table 4) (S2 Fig).

**Table 4:** Pearson´s correlations between morphometric measurements and colouration of the combined *Agalychnis* spp. data from Costa Rica.

Parameter	Weight	Length	Ratio
**Dorsal**			
L*	**-0.480**†	**-0.364**†	**-0.423**†
-a*	0.006	-0.051	0.038
+b*	**-0.383**†	**-0.282**†	**-0.347**†
**Chroma**	**-0.360**†	**-0.248**†	**-0.337**†
**Hue**	**0.337**†	**0.295**†	**0.272**†
**Ventral**			
L*	**-0.509**†	**-0.266**†	**-0.550**†
+a*	**0.407**†	0.161	**0.478**†
+b*	-0.138	**-0.374**†	0.058
**Chroma**	-0.088	**-0.333**†	0.103
**Hue**	**-0.547**†	**-0.315**†	**-0.574**†

^**†**^Significant (p<0.05). Colour intensity in the table indicates the strength of the relationship between variables.

L: lightness,

-a*: green coordinate,

+b*: yellow coordinate

The relationship between hue and body condition of the animals varied inversely, depending on location measured (back or belly), and is stronger in the case of the ventral colour.

#### Blood values and colouration

Glucose showed a positive relationship with ventral lightness, chroma as well as +a* and +b*values of colour and a negative relationship with hue (p<0.0001). Meanwhile, protein was more weakly correlated to ventral +b* coordinates and chroma (p = 0.01). Haematocrit also had significant relationships (p = 0.003) with all the colour values except the dorsal –a* coordinate, ventral +b*, and chroma (Table 5) (S2 Fig).

**Table 5:** Pearson´s correlations between blood values and colouration of the combined *Agalychnis* spp. data from Costa Rica.

Parameter	Glucose	Protein	Haematocrit
**Dorsal**			
L*	0.055	-0.035	**-0.356**†
-a*	-0.115	-0.191	-0.031
+b*	-0.017	-0.050	**-0.394**†
**Chroma**	-0.003	-0.008	**-0.355**†
**Hue**	0.081	0.229	**0.443**†
**Ventral**			
L*	**-0.449**†	-0.244	**-0.429**†
+a*	**0.463**†	0.263	**0.393**†
+b*	**0.523**†	**0.359**†	0.083
**Chroma**	**0.529**†	**0.362**†	0.128
**Hue**	**-0.361**†	-0.232	**-0.478**†

^**†**^Significant (p<0.05). Colour intensity in the table indicates the strength of the relation between variables.

L: lightness,

-a*: green coordinate,

+b*: yellow coordinate

Colours (except hue) on the back of the frogs (dorsal) were all inversely related to the haematocrit values. Glucose and haematocrit both showed an inverse correlation with the lightness and hue of the ventral colours, whereas glucose varied positively with ventral +a* and +b* values as well as chroma.

The results suggest an important relationship among several blood parameters, colouration and body condition of the tree frogs, and that these integrated parameters may all be considered in describing nutritional status of the *Agalychnis* spp. frogs.

## Discussion

This study demonstrated clear links among body condition in tree frogs in the wild, their skin colour traits and their blood parameters; physiological biomarkers of nutritional status showed associations with skin colour that were not reflected in body condition. Additionally, several differences in variables between populations were described.

### Differences among species/locales

Apart from differences among species, we found in this study contrasts between animals from both populations of *A*. *callidryas*. Length (49–52 mm) values shown by both groups were similar compared to those reported in several studies (46–53 mm [57], 45–46 mm [58]) for free-range males, however, weights were higher in frogs from this study compared to others [57]. *A*. *annae* length (average 58 mm) was lower than data previously reported (64 mm and 74 mm) [59,60], but the body condition (ratio weight/length) was higher than *A*. *callidryas*. The variation in body condition responds to several factors; it can reflect increased availability of food resources as well as adequate nutrition, and therefore provide a positive indicator of reproductive success [61]. Likewise, body condition is also related to environmental changes which disturb the habitat’s normal conditions [31], the pattern of predation [58] and/or the timing of the breeding season [62]. Body condition and the factors affecting that parameter have been not studied in *A*. *annae*.

Glucose values found in this study were similar to those reported for *Rana catesbeiana* in captivity and free-range *Polypedates teraiensis* (38–53 mg/dl) [33,41,63,64], although *A*. *callidryas* from the Pacific showed slightly higher values. The blood glucose concentration is the result of the rates of entry and removal in the circulation, which can be influenced by several variables including the supply of nutrients and hormonal interactions as well as the regulatory mechanisms of the liver [36,41]. The “normal” values seen here, combined with adequate body condition, suggest that frogs were at least feeding, thus had food resources in their habitat.

Plasmatic protein values were slightly lower than values of 4.1 to 11.8 g/dl reported in *R*. *catesbeiana* in captivity [63,64], *Polypedates teraiensis* [33] and free-range *Pseudepidalea viridis*, *Pelobates syriacus*, *Rana dalmatina*, *Hyla arborea* and *Pelophylax ridibundus* [43]. In carnivores, glucogenic aminoacids can influence glucose synthetized from gluconeogenesis and its utilization [36]. Likewise, the circulating protein levels are tightly related to the dietary protein content and its utilization efficiency [65]. Data here suggest that food, while available, may have varied in protein quality.

Haematocrit values of all groups were similar to those found for several anurans, within a range of 22.4 to 58.5% [33,43,64,66,67], which is indicative of adequate hydration status. Changes in haematocrit have been shown as a consequence of the alimentary status of the animal, the supply of specific minerals (iron, copper and selenium) in addition to dietary protein, folic acid, cobalamin and niacin. In various studies, haematocrit has been shown to be affected by environmental conditions as well as the affinity for oxygen by the haemoglobin (oxidative status), depending on the species [36,40,63,64]. *A*. *annae* showed the highest haematocrit level; high levels are often related with dehydration in small animals [68]. However, no other plasma measures differed significantly in *A*. *annae*, suggesting that hydration status alone may not underlie the haematocrit values seen.

The three groups from the study showed variation in colour measurements. Atlantic and Pacific populations of *A*. *callidryas* exhibit a highly localized variation in colour pattern, supporting the role of biogeographical barriers to gene exchange [52]. Nonetheless, colour diversity and fluctuation in amphibians is also influenced by multiple factors including diet, protection against predators, water balance, thermal regulation, temperature, solar radiation, light intensity and other biotic aspects, to which the animals are exposed simultaneously [23,69].

In addition to differences found between populations, individual variations can also occur, although such mechanisms remain poorly understood [70]. There have been suggested intrinsic variations in body size in *Pseudophryne corroboree* [70], glucose levels in *Rana sylvatica* [71] and colouration in *Dendrobates auratus* [72]; however, in order to monitor these changes it is necessary to keep the animals in captivity, and in this study the animals were measured only once. In this regard, more research is necessary to confirm the true nature of those underpinning variations.

### Associations between parameters of the combined *Agalychnis* spp. data from Costa Rica

#### Blood values and body condition

Indicators for protein status such as plasmatic proteins and haematocrit can be linked with body condition, given that they function as transporters of vitamins, lipids and minerals, and some are also indicators of protein biosynthesis [36,73]. Several studies have demonstrated that plasmatic proteins can reflect the quality and quantity of dietary proteins in *R*. *catesbeiana* [63], which also show good responses in terms of growth rate, weight gain and general body condition with high levels of dietary proteinin both *Rana catesbeiana* [65] and *R*. *rugulosa* [74]. We thus suggest that these good food resource indicators imply that were adequate nutrients available for the animals in these habitats, utilized to improve body condition.

Diets of carnivores normally have low carbohydrate content; the activities of enzymes related to downstream glucose metabolism such as hepatic glucokinase, fructokinase and glycogen synthase are very weak [38,75]. Gluconeogenesis instead utilizes certain dietary aminoacids to supply the glucose need [36,38]. In anurans mainly surviving on insect or other animal food resources, the “carnivore” gluconeogenic pathway therefore explains why plasma protein and blood glucose concentrations correlate well. The condition of the animal may reflect the pool of available energy needed for body maintenance [57,76]. Therefore, the energy status and the body condition of the animals under natural circumstances is determined by the capacity to produce glucose, clarifying the observed correlations between body condition on the one hand, and blood glucose and plasma protein concentrations on the other hand.

Body condition also showed important positive associations with haematocrit values. The latter are closely related with erythrocyte counts, as well as haemoglobin and food intake in *Rana tigrina* [66], and with body size and weights of the mountain chicken frog *Leptodactylus fallax* and the chorus frog *Pseudacristriseriata* [77,78]. Haemoglobin synthesis involves different pathways through red blood cell precursors and reticulocytes, globin chains and *heme* groups as well as iron as intermediates, all of them directly or indirectly linked to aminoacids and therefore with protein activity [36,79]. Haematocrit determination thus can be an indicator of both body condition and erythropoietic status in the tree frogs, and consequently reflect the nutritional condition of the animals, but more specifically is related to protein nutrition than body condition *per se*.

#### Body condition and colouration

In addition to the variations of physical appearance as signals of crypsis or aposematism, mating success and sexual selection are also affected by the visual perception of the mate [80]. Moreover, some authors have related reptiles and amphibian males´ colouration directly with reproductive behaviour [17,32,52,81], therefore, the analysis of the colour associations will be focused mainly in that direction.

Despite the non-specific character of body condition, frogs in better condition did differ in skin colouration, displaying darker and less saturated colours in both backs and bellies, as well as less colour intensity in the belly. Consequently, small males can display brighter and more saturated and intense colours in both dorsally and ventrally. Although colouration did not vary with body size in male moor frogs (*Rana arvalis*), within small males the bluer ones were more successful in obtaining a mate [32], while large body size was not identified as a sexually selected trait in male red-spotted newts (*Notophthalmus v*. *viridescens*) [17]. Likewise, no effect of male´s body size on female choice was demonstrated in the red-eye frog *A*. *callidryas* from both Pacific and Atlantic populations [51], and large size males did not have an advantage in mating success in the serrate-legged small tree frogs *Philautus odontotarsus* [82].

Belly colour in males may be essential due to its influence on colour of the vocal sac, which plays a critical reproductive function [21]. Females of the European tree frog (*Hyla arborea*) prefer males showing vocal sacs with more saturated and intense colours under nocturnal conditions [18], while female red-eye frogs choose males in the absence of acoustic signals, indicating that other cues could play more suitable roles in female choice [51]. Although mating calls have been reported from *A*. *annae*´s males [60], relationships among vocal sacs (acoustic or visual cues), colour and mating choice have not yet been determined for this species.

The inverse association found between colour and body condition suggests that smaller males may invest more effort in pigment mobilization towards their skin to look brighter with more intense colours in order to be chosen by the females. However, this response is also linked with the perception of the mate, where vision plays an important role in anuran sensing [21]. Frog vision is characterized by the presence of two types of rods (green and red) which allow them to discriminate the brightness as well as the colours [83], so they can use colour vision for mate recognition in dim light [84]. Nevertheless, the visual communication of colour in *Agalychnis* species is beyond the scope of this study.

Results revealed that the size of males can indicate how they express their colouration, and in some cases, perhaps colouration is a better trait for mate selection than body size. Furthermore the capacity of males to afford dietary carotenoid mobilization to the skin, and all the metabolic pathways involved in such mechanisms [11], suggest that body “condition” should be considered multifactorial beyond simply size and weight. Rather, the capacity to maintain optimal functionality and essential cellular processes is an additional critical component. Although ornament production and signalling have a direct intrinsic connection with adequate performance of the organism, these parameters together could be a more valuable signal to female mate choice [85]. Both ornament traits as well as condition are directly linked with nutrition in several animal species [86,87]. Dietary carotenoids could thus induce colour saturation by an increasing of the chroma in males during the breeding season, no matter their size, to attract mating females and signal more quality and suitable nutritional status.

#### Blood values and colouration

Glucose levels were significantly correlated with ventral, but not dorsal, colours of the animals. At high glucose levels, orange and yellow colours of the frogs looked darker, less intense and more saturated, whereas at low levels colours displayed lighter and more intense but less saturated. Glucose might influence colouration in terms of energetics. Some authors have demonstrated that metabolism of pigments and carotenoids involves an energetic cost to the animal, including the conversion of precursors to the pigments deposited in the feathers or skin, absorption, transport and deposition [11,88].

Plasma protein level was significantly correlated with the ventral yellow coordinate as well as the chroma of the frogs, meaning that the animals with high levels of protein displayed yellower saturated colours. As plasmatic proteins can reflect general nutritional status of the animals [11,65], male frogs with good food sources are likely capable to display more saturated colours in their bellies. Glucose as well as glucogenic aminoacids (Alanine, Serine, Glycine, Aspartate, Glutamate) [89], as a source of energy entering into the cytoplasm and mitochondria for cellular respiration and ATP synthesis, can directly affect ornamental traits of the animals, in terms of the energy demanded and the capacity of energy utilization for ornament production [76]. We thus suggest that colouration can signal energy status of the animals, reflected in the circulating glucose and protein concentrations in the blood. Carotenoids can have a role in energy production through their oxidation in the inner membrane of the mitochondria [90]. It is therefore probable that only male frogs with adequate quantities of carotenoids can afford this function and at the same time display different patterns of colour saturation and/or intensity in their belly.

Haematocrit was also correlated with both dorsal and ventral colours. Dorsally, it seems that changes in this parameter inversely affect the lightness, yellow coordinate and chroma, and directly affects the intensity of the colour. At the ventral level, the higher the haematocrit, the darker and less intense the colours, although there is a positive relation with the red coordinate. Haematocrit signals both erythrocyte and haemoglobin synthesis and function, using glucose as the primary substrate for energy needs in several domestic species [36,66]. The *heme* group is synthetized within the mitochondria involving Krebs cycle intermediates as substrates. Red blood cells also require energy in the form of ATP for maintenance of shape, phosphorylation of membranes, phospholipids and proteins, transport of various molecules and partial synthesis of purine and pyrimidine nucleotides, among others functions [36,91]. We assume that glucose supply affects haematocrit values as a signal of red blood cell status and erythropoiesis, and can be reflected in the colouration of the animals, given that only healthy animals display a striking ornamentation. However, specific pathways of blood cell metabolism need to be further studied in amphibian models.

In conclusion, this study in wild male tree frogs demonstrates that associations among blood parameters, body condition and colouration can be used to assess nutritional status in the animals. While we recognize potential limitations of comparing populations over two different years, these findings are considered a foundation study. Given that there is littleinformation available regarding blood biochemistry for the *Agalychnis* species, the values obtained could be seen as general reference values for natural populations. Particular skin colouration traits vary with body condition and size, while blood metabolites show additional associations with skin colour that is not all reflected by body condition, and indicates that body condition in terms of weight and length does not represent a complete image of fitness. However, when using colouration and blood values as markers for nutritional status, one needs to take into account potential differences between populations, as observed between the two locations of *A*. *callidryas* in this study. These differences could be influenced by several circumstances, like nutritional composition of prey, availability of foods for the prey, differences in weather conditions as well as specific ecosystem biodiversity; further studies are required to support these statements. Finally, these data appear to represent the normal status of the animals in their native environment. These results, obtained under natural conditions, can thus be used as baselines to evaluate the impact of diets as well as the quality of the ingredients used in feeding captive *Agalychnis* spp.

## Supporting information

S1 Fig Dorsal (D) and ventral (V) colour in three populations of *Agalychnis* sp. Z: Atlantic population, SI: Pacific population, a and b: coordinates of the chromaticity diagram, L: lightness, c: chroma, h: hue.
(PDF)

S2 Fig Matrix of associations between blood metabolites, body condition and colouration of the combined *Agalychnis* spp. data from Costa Rica. (PDF)
