# Supplementary material for: Colouration in amphibians as a reflection of nutritional status: The case of tree frogs in Costa Rica
Source: PLoS One. 2017 Aug 24;12(8):e0182020. doi: 10.1371/journal.pone.0182020 (PMC5570269; doi:10.1371/journal.pone.0182020)
Supplement: S1 Fig — Z: Atlantic population, SI: Pacific population, a and b: coordinates of the chromaticity diagram, L: lightness, c: chroma, h: hue. (PDF) [file pone.0182020.s001.pdf]

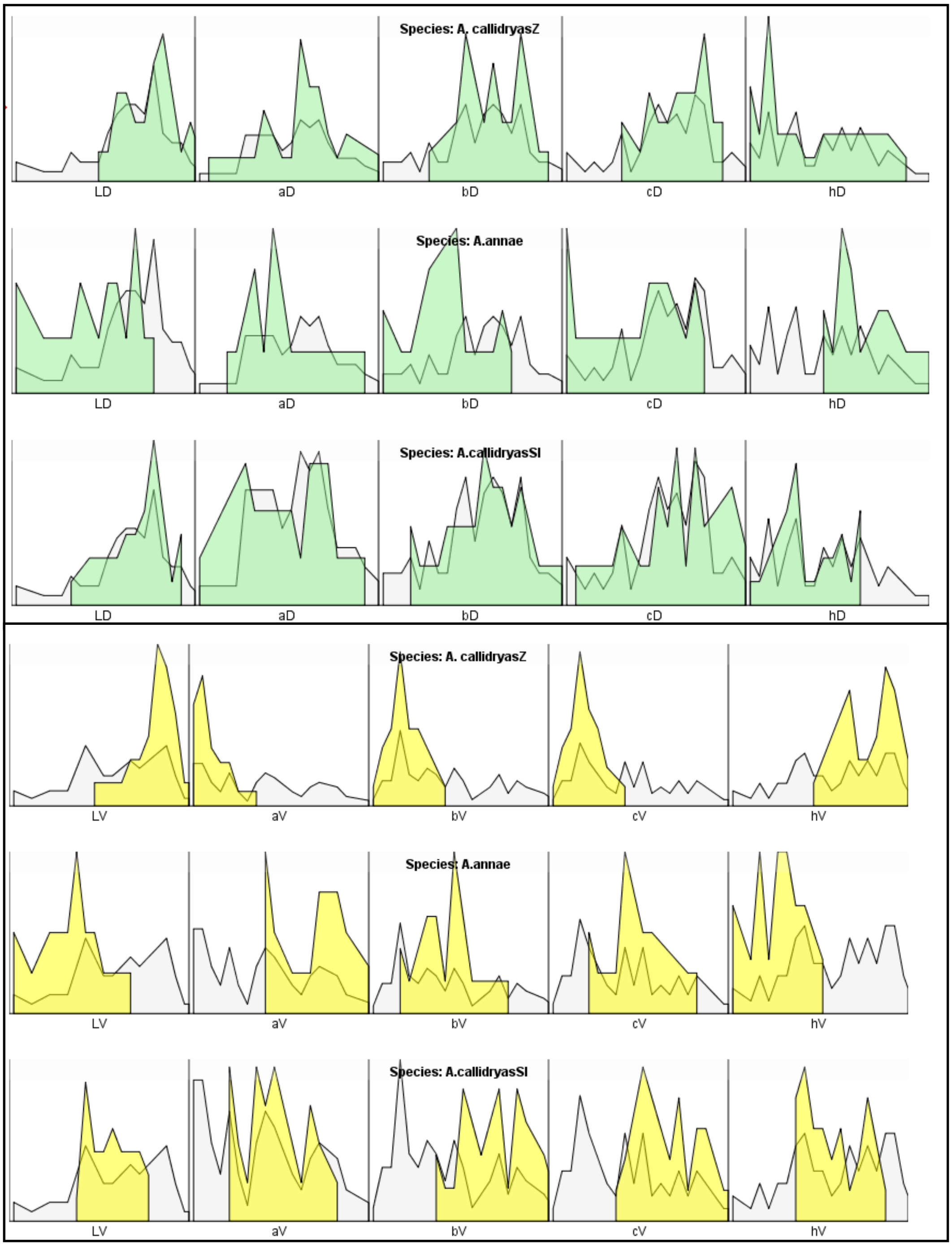

S1. Dorsal (D) and ventral (V) colour in three populations of *Agalychnis* sp.
Z: Atlantic population, SI: Pacific population, a and b: coordinates of the chromaticity diagram, L: lightness, c: chroma, h: hue.
