# Supplementary material for: Colouration in amphibians as a reflection of nutritional status: The case of tree frogs in Costa Rica
Source: PLoS One. 2017 Aug 24;12(8):e0182020. doi: 10.1371/journal.pone.0182020 (PMC5570269; doi:10.1371/journal.pone.0182020)
Supplement: S2 Fig — (PDF) [file pone.0182020.s002.pdf]

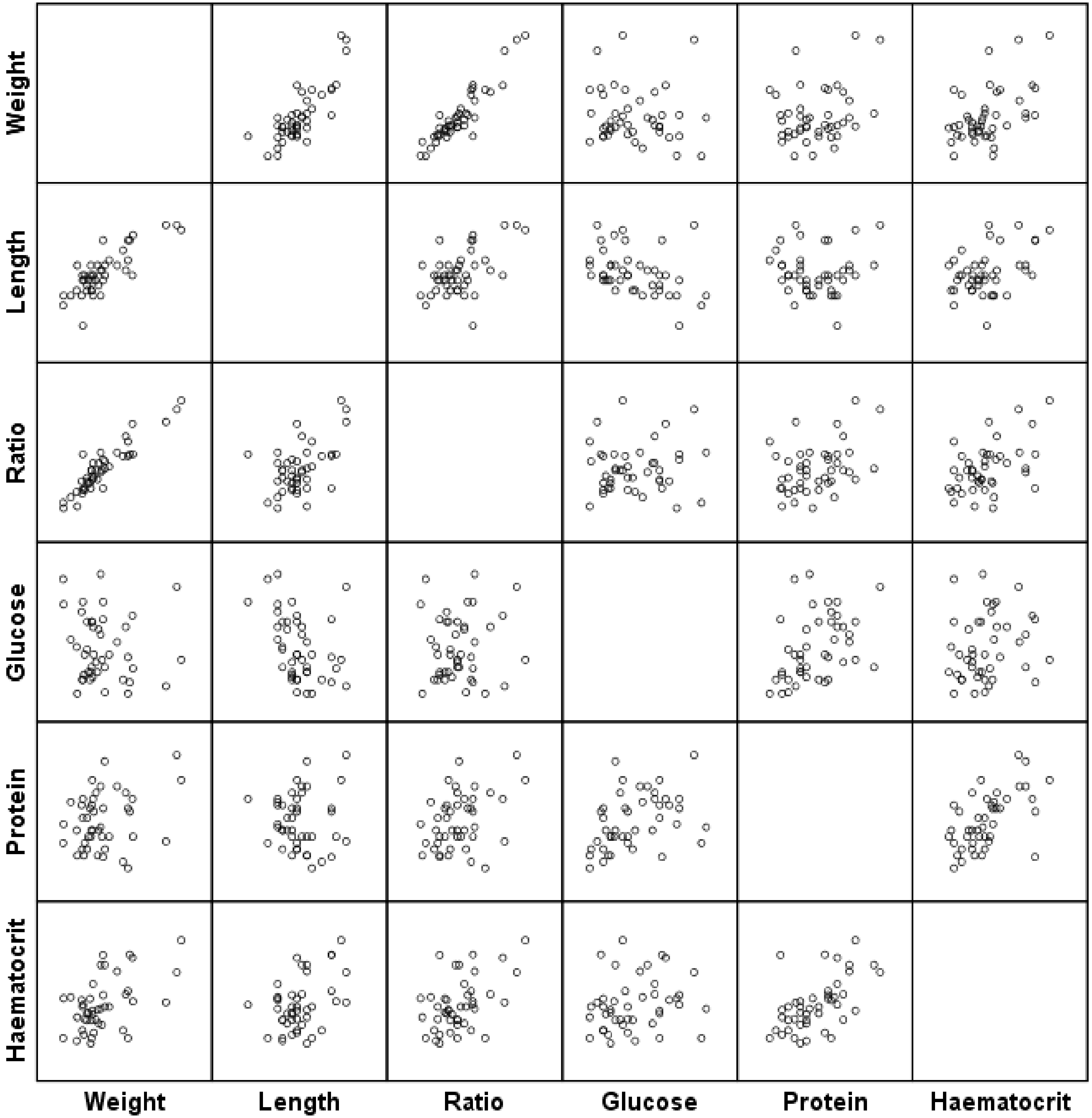

S2A Fig. Matrix of associations between blood metabolites and body condition of the combined *Agalychnis* spp. data from Costa Rica.

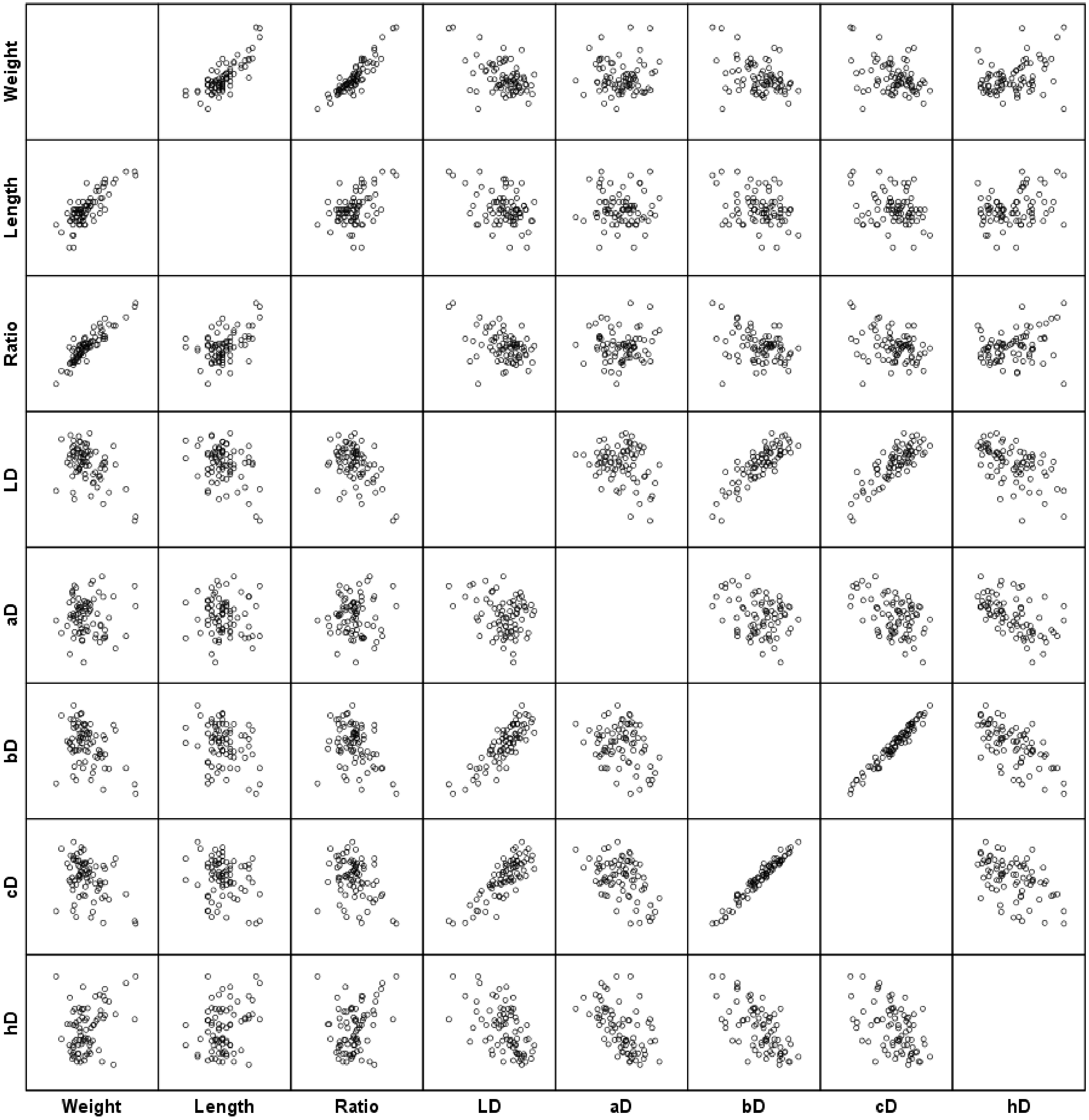

S2B Fig. Matrix of associations between body condition and dorsal colouration of the combined *Agalychnis* spp. data from Costa Rica.

.

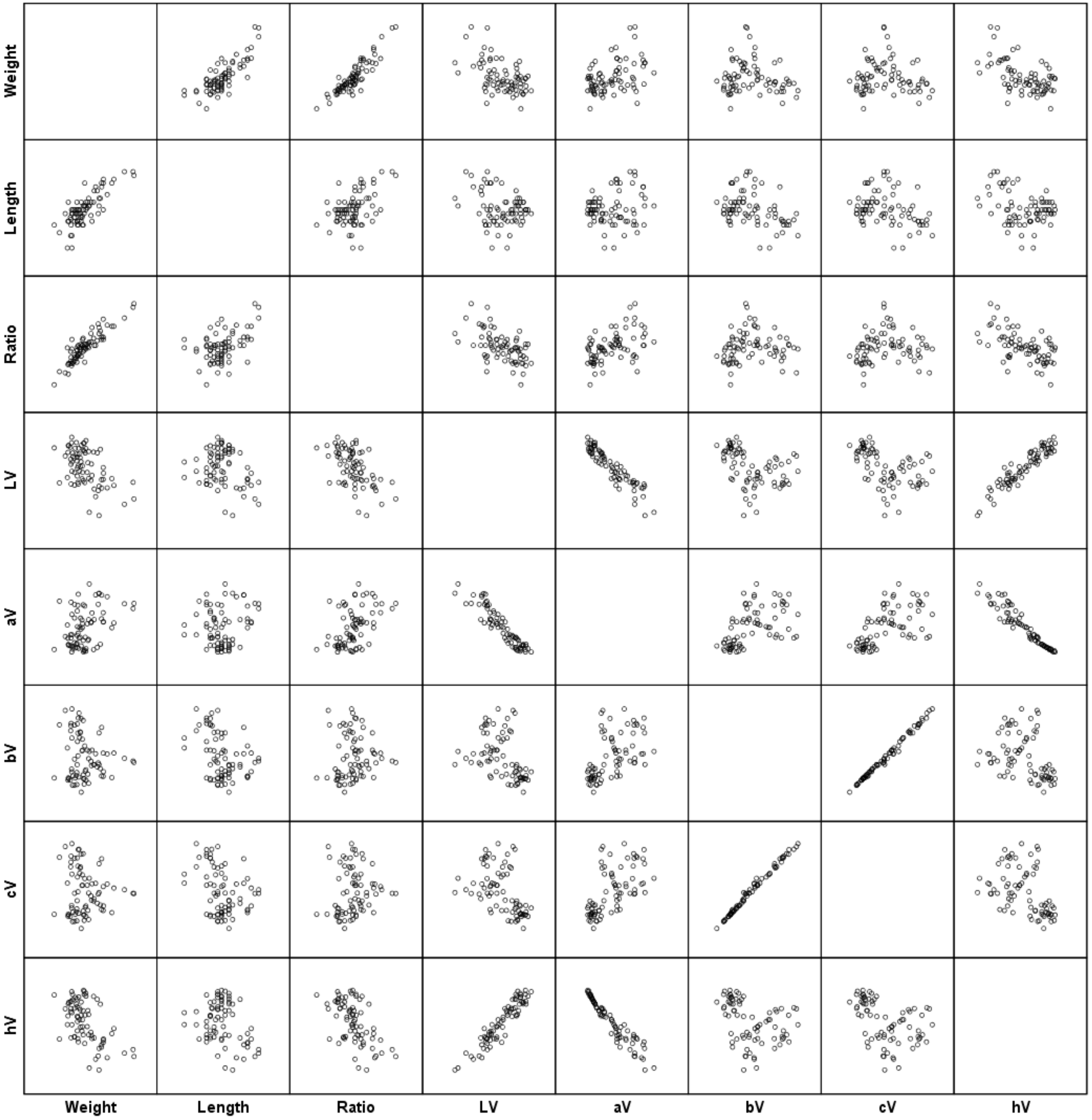

S2C Fig. Matrix of associations between body condition and ventral colouration of the combined *Agalychnis* spp. data from Costa Rica.

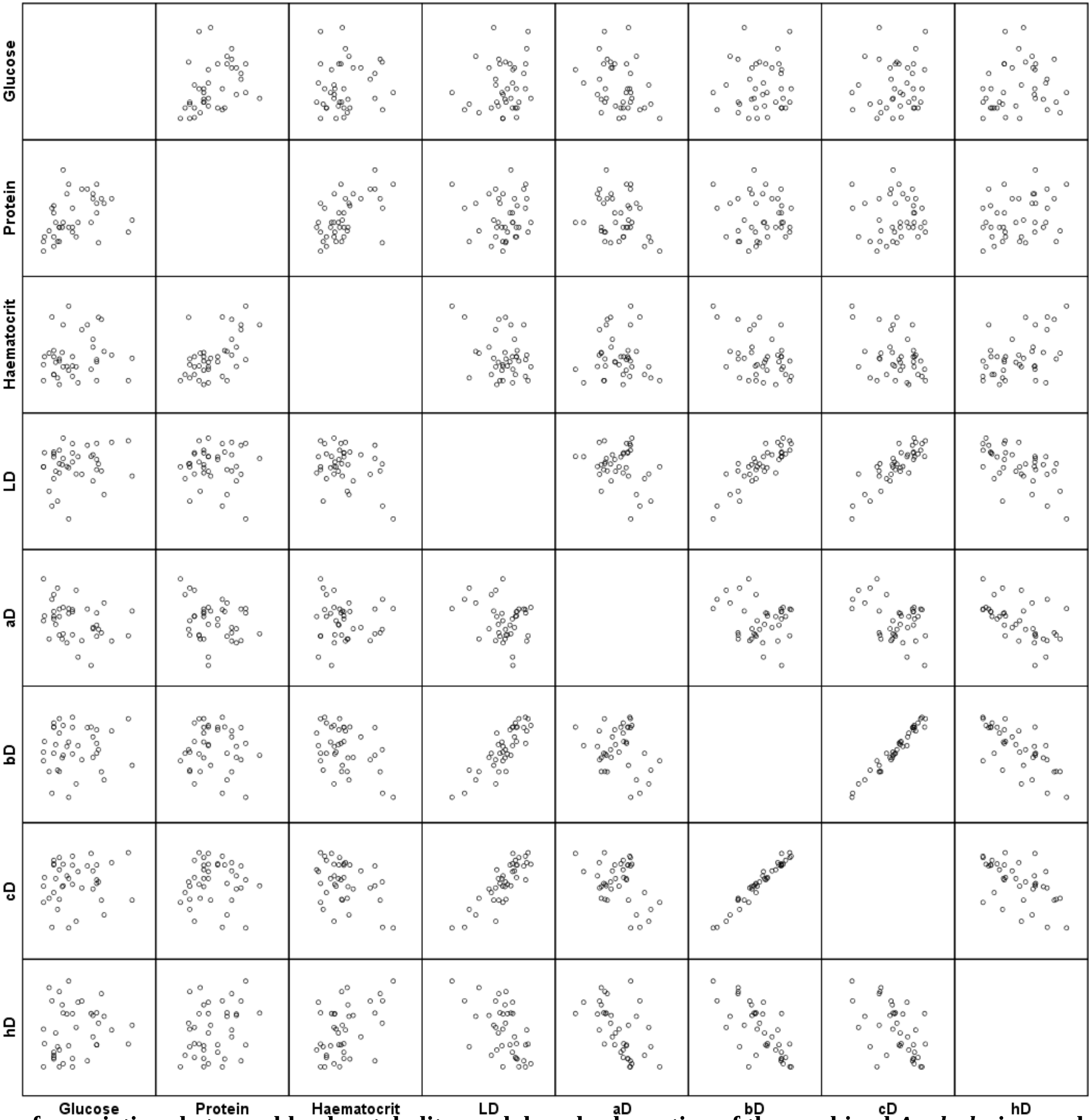

S2D Fig. Matrix of associations between blood metabolites and dorsal colouration of the combined *Agalychnis* spp. data from Costa Rica.

.

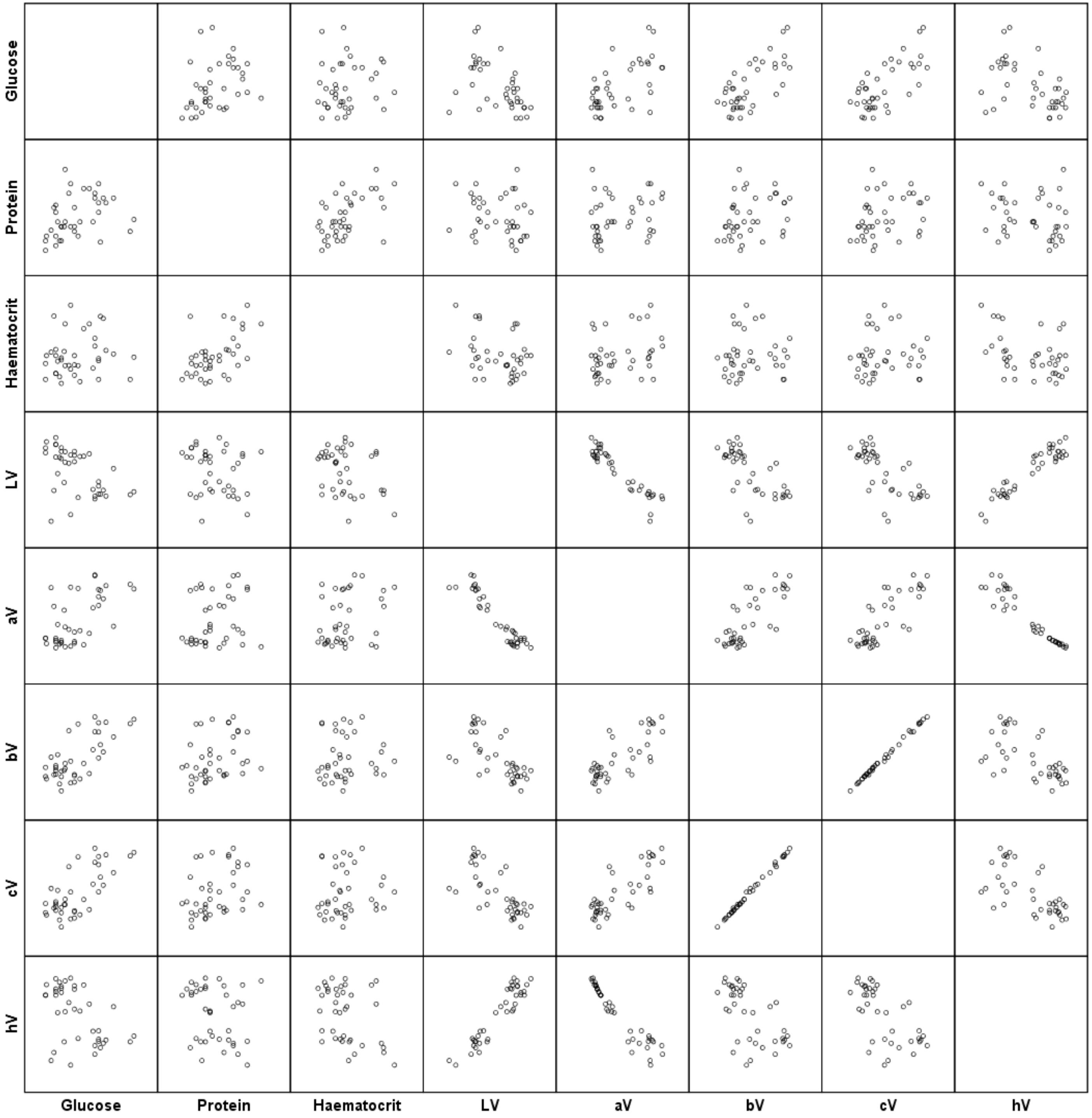

S2E Fig. Matrix of associations between blood metabolites and ventral colouration of the combined *Agalychnis* spp. data from Costa Rica.
